# Deep learning for predicting future lesion emergence in high-risk breast MRI screening: a feasibility study

**DOI:** 10.1186/s41747-023-00343-y

**Published:** 2023-06-07

**Authors:** Bianca Burger, Maria Bernathova, Philipp Seeböck, Christian F. Singer, Thomas H. Helbich, Georg Langs

**Affiliations:** 1 Department of Biomedical Imaging and Image-Guided Therapy, Division of Computational Imaging Research (CIR), Medical University of Vienna, Währinger Gürtel 18-20, 1090 Vienna, Austria; 2 Department of Biomedical Imaging and Image-Guided Therapy, Division of General and Pediatric Radiology, Medical University of Vienna, Vienna, Austria; 3 Department of Obstetrics and Gynecology, Division of Special Gynecology, Medical University of Vienna, Vienna, Austria; 4 Comprehensive Cancer Center, Medical University of Vienna, Vienna, Austria; 5 Computer Science and Artificial Intelligence Laboratory, Massachusetts Institute of Technology, Cambridge, MA USA

**Keywords:** Breast cancer, High-risk women, Magnetic resonance imaging, Deep learning, Generative adversarial network

## Abstract

**Background:**

International societies have issued guidelines for high-risk breast cancer (BC) screening, recommending contrast-enhanced magnetic resonance imaging (CE-MRI) of the breast as a supplemental diagnostic tool. In our study, we tested the applicability of deep learning-based anomaly detection to identify anomalous changes in negative breast CE-MRI screens associated with future lesion emergence.

**Methods:**

In this prospective study, we trained a generative adversarial network on dynamic CE-MRI of 33 high-risk women who participated in a screening program but did not develop BC. We defined an anomaly score as the deviation of an observed CE-MRI scan from the model of normal breast tissue variability. We evaluated the anomaly score’s association with future lesion emergence on the level of local image patches (104,531 normal patches, 455 patches of future lesion location) and entire CE-MRI exams (21 normal, 20 with future lesion). Associations were analyzed by receiver operating characteristic (ROC) curves on the patch level and logistic regression on the examination level.

**Results:**

The local anomaly score on image patches was a good predictor for future lesion emergence (area under the ROC curve 0.804). An exam-level summary score was significantly associated with the emergence of lesions at any location at a later time point (*p* = 0.045).

**Conclusions:**

Breast cancer lesions are associated with anomalous appearance changes in breast CE-MRI occurring before the lesion emerges in high-risk women. These early image signatures are detectable and may be a basis for adjusting individual BC risk and personalized screening.

**Relevance statement:**

Anomalies in screening MRI preceding lesion emergence in women at high-risk of breast cancer may inform individualized screening and intervention strategies.

**Key points:**

• Breast lesions are associated with preceding anomalies in CE-MRI of high-risk women.

• Deep learning-based anomaly detection can help to adjust risk assessment for future lesions.

• An appearance anomaly score may be used for adjusting screening interval times.

**Graphical Abstract:**

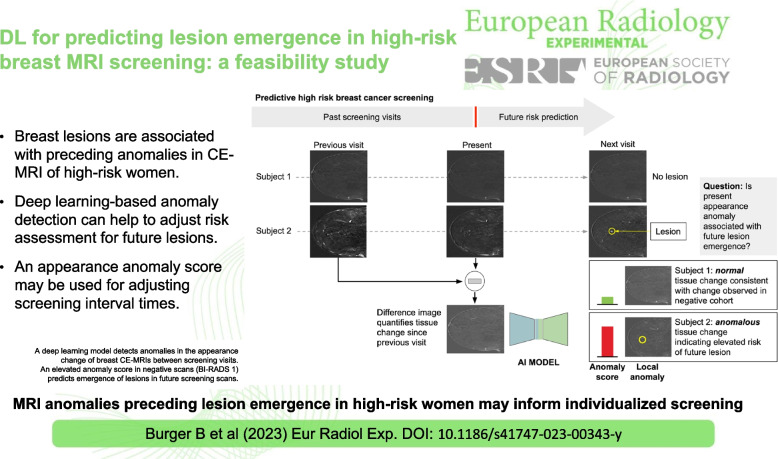

**Supplementary Information:**

The online version contains supplementary material available at 10.1186/s41747-023-00343-y.

## Background

Up to 20% of BCs occur in women with a genetic predisposition [1]. To achieve a significant risk reduction, these women may opt for a prophylactic bilateral mastectomy or are offered intensified annual screening [2, 3]. Although mammography is sufficient in some women [4], international societies have issued guidelines for high-risk BC screening, recommending contrast-enhanced magnetic resonance imaging (CE-MRI) of the breast as a supplemental diagnostic tool due to its high sensitivity [5–7]. Given limited resources, the effectiveness and feasibility of screening programs depend on the balance between early detection capability and over-screening.

Clinical risk models rely on static categorical variables including patient demographics, personal and family history, and risk factors driven by the patient’s age [8], resulting in the inclusion of women into rigid screening regimes. Although CE-MRI improves screening sensitivity significantly, cases of invasive BC can be missed, or cancer can develop between screens as interval BC [9, 10]. In hindsight, about a third of cancers detected in high-risk screening programs had been already visible at the last negative CE-MRI screen, indicating missed cancers by reporting radiologists despite improved training [11–13]. This points to the opportunity to exploit even negative screening CE-MRI exams for risk assessment enabling dynamic screening regimes.

Here, deep learning (DL) models may detect precursor image signatures in CE-MRI associated with an increased risk of future lesion emergence. Image-based individual short- to mid-term risk adjustment and corresponding optimization of screening intervals could enable continual personalization of screening programs leading to improved screening effectiveness [14–17].

DL has been widely implemented in healthcare for pattern recognition, including radiology [18, 19]. In breast imaging, DL systems have been quickly evolving, surpassing the performance and clinical value of traditional computer-aided detection systems for mammography [20] or dynamic CE-MRI [21]. Supervised models detect lesions [22] by either parsing imaging data [23] or analyzing entire MRI slices at once [24, 25]. Furthermore, DL may reduce workload by being trained to triage negative screens [26, 27], which would allow radiologists to concentrate only on suspicious scans. DL-based prediction models have also been proposed to predict treatment response or assess the overall risk of cancer [28–30]. In the context of breast cancer, initial results using mammography of women at normal risk suggest the feasibility to predict certain pathological conditions in the future [14–16]. However, to our knowledge, dynamic risk assessment based on CE-MRI of high-risk women has not been explored yet.

In this study, we investigated two questions: (1) Can DL models detect “anomalies” as deviations from normal tissue changes in negative CE-MRI screens of the breast of high-risk women? (2) Are these automatically detected deviations to which we refer to as anomalies associated with future lesion emergence?

## Methods

### Study overview

**Fig. 1:**
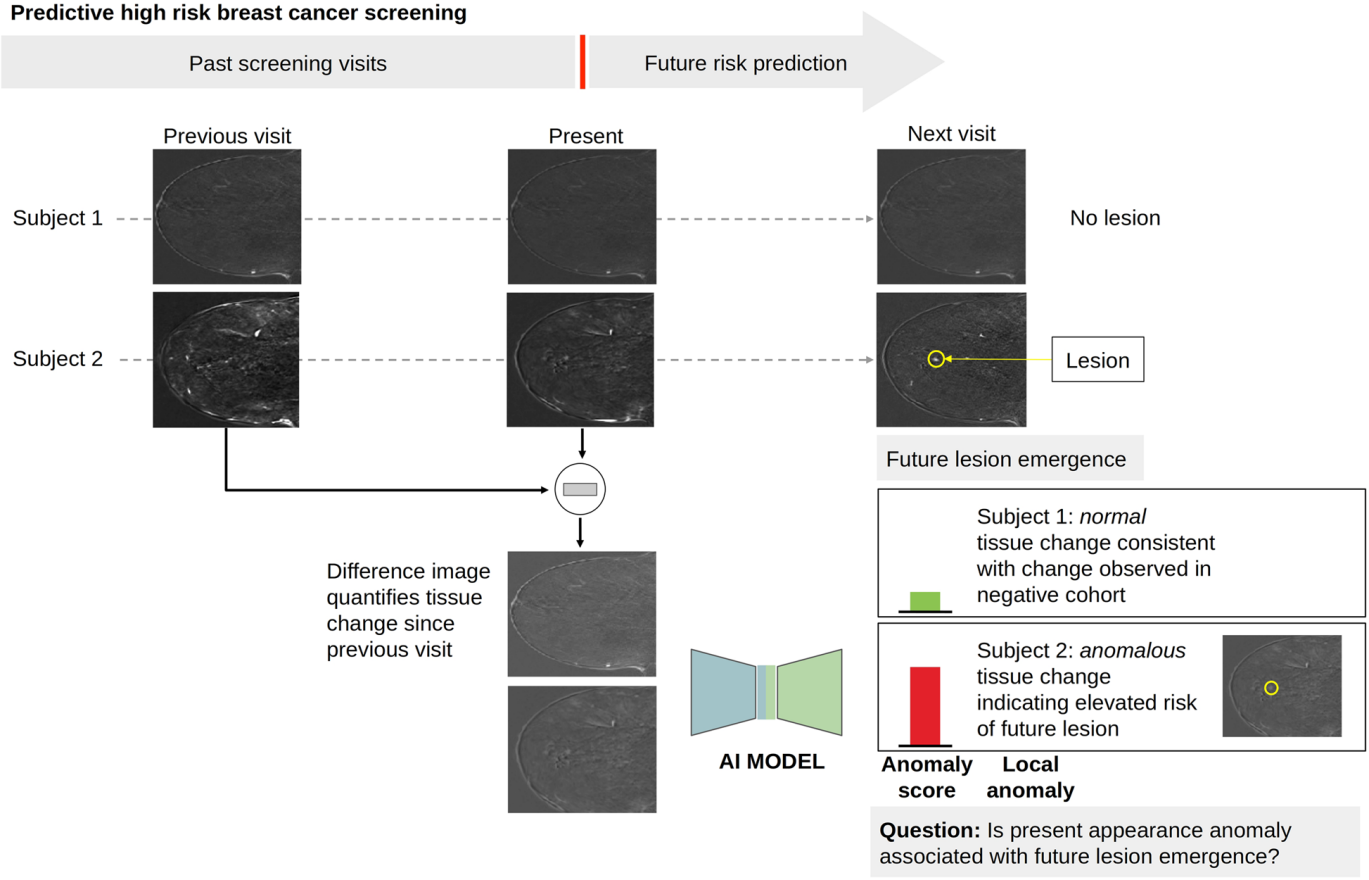
Study overview for identifying anomalies in screening CE-MRI of the breast associated with future lesions. A deep learning model detects anomalies in the appearance change of breast CE-MRIs between screening visits. An elevated anomaly score in negative scans (BI-RADS 1) predicts the emergence of lesions in future screening scans. Anomalies may serve as a basis for dynamic risk profiling in breast cancer screening to optimize individual screening intervals. *BI-RADS* Breast Imaging and Reporting Data System, *CE-MRI* Contrast-enhanced magnetic resonance imaging

The ratio of positive to negative cases in screening cohorts is low, and the precursor signatures of lesions are not known. For an automated approach to dynamic risk profiling the negative cohort could therefore serve as a training cohort to create a model that becomes sensitive to deviations from normal variability in new cases and identifies them as anomalies. To answer our research questions, we therefore first built a model capturing the variability of breast tissue in healthy women between screening visits. New CE-MRI examinations were then assessed by comparing their appearance with the normal appearance model. Deviations from the model of normal variability were thereby captured by an anomaly score (Fig. 1). Since the detected anomalies might serve as a basis for dynamic risk profiling, their association with future lesions was evaluated.

### Data collection

This study on prospectively collected data was approved by our institutional ethics committee. We analyzed anonymized patient data of subjects who gave informed consent for inclusion. We collected longitudinal dynamic CE-MRI scans of the breast from 1,119 high-risk women with a lifetime risk for breast cancer higher than 20%, who underwent high-risk screening at our institution from 2002 to 2019. Studies of women with unilateral or bilateral mastectomy or with implant reconstruction were excluded. Subjects whose studies were acquired exclusively until 2007 were also excluded. 2007 was chosen as the cutoff due to a scanner switch becoming effective at the beginning of 2008. We identified subjects for whom at least three imaging exams were available, of which the first two were BI-RADS 1. This resulted in a set of 96 subjects, 63 subjects with three BI-RADS 1 exams forming the negative cohort, and 23 subjects with two BI-RADS 1 exams and a subsequent BI-RADS 2, 3, 4, or 5 exams forming the positive cohort. BI-RADS 2 and 3 lesions were also included in the positive cohort since we were interested in differentiating normal tissue from any type of lesion.

**Fig. 2:**
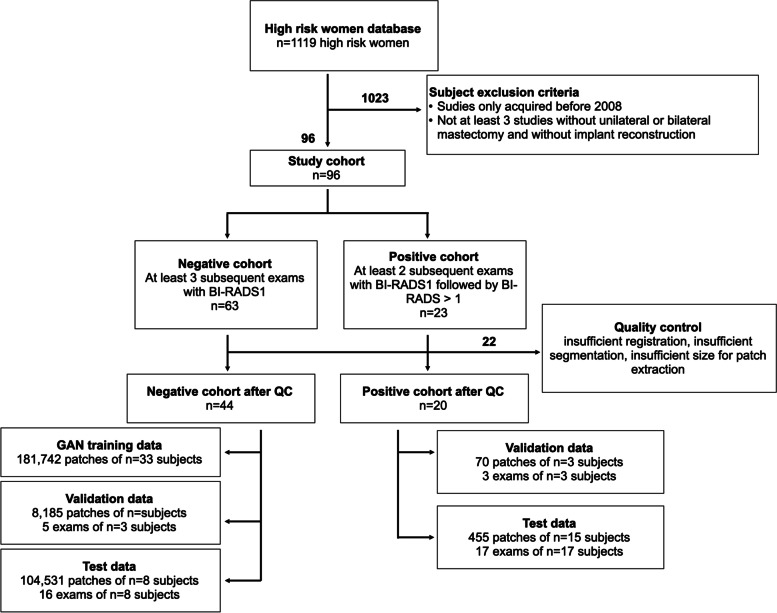
Overview of dataset collection and use

We conducted preprocessing, including intra-patient registration, breast segmentation, and patch extraction (more details in the “Preprocessing” section and Supplementary Material). Subjects with unsatisfactory registration or segmentation results or a breast size too small to extract patches of the desired size were excluded. The final dataset therefore comprised 44 women in the negative cohort and 20 women in the positive cohort. Figure 2 gives an overview of the creation of our dataset.

### MRI acquisition

During the study period, two scanners and corresponding protocols were in use. We only summarize the most important information for this study. A more detailed description of imaging protocols can be found in Riedl et al. [31, 32] and Milos et al. [33]. From 2002 to 2007 (Period 1), scans were performed with a 1.0-T scanner (Gyroscan T10-NT, Philips, Amsterdam, the Netherlands). T1-weighted three-dimensional gradient-echo dynamic sequences were obtained once before and 6 times after the injection of a single dose of a gadolinium-based contrast agent (Dotarem (gadoteric acid) 0.1 mmL/kg, rate 3 mL/s with 20 mL saline flash) at intervals of 70 s. For each image, 30–36 axial slices with a spacing between slices of 4.0–4.5 mm and a matrix of 256 × 256 (1.25–1.48 mm isotropic) were done. From 2008 to 2019, a 1.5-T MRI scanner Magnetom Avanto (Siemens, Berlin, Germany) was used. From 2008 to 2013 (Period 2), T1-weighted dynamic sequences were acquired once before and four times after the injection of the contrast agent at intervals of 90 s. For each image, 48–54 axial slices with a spacing between slices of 3.30–3.85 mm and a matrix of 384 × 384 (0.91–0.99 mm isotropic) were done. From 2014 onwards (Period 3), the imaging protocol was updated and the axial dynamics were changed to a higher spatial resolution, Dixon fat-suppressed VIBE (volumetric interpolated breath-hold examination) sequence measured once before and three times after contrast agent injection at intervals of 90 s. From 80 to 88 axial slices with spacing between slices of 2.00 mm and a matrix of 512 × 512 (0.70–0.74 mm isotropic) were acquired.

### Data preparation

The negative cohort was randomly split into a training (75%), validation (7%), and test set (18%), while the positive cohort was randomly split into a validation (15%) and test set (85%). There was no overlap of patients between sets. The training set consisted of MRI data of 33 women of the negative cohort from which 181,742 patches covering breast tissue were randomly extracted (more details on patch extraction in the “Preprocessing” section and Supplementary Material). The validation set was formed of 3 women from the negative cohort and 3 from the positive cohort, with corresponding randomly sampled 8,185 normal patches and 70 patches from locations with future lesions. The test set consisted of 104,531 randomly sampled normal patches extracted from 8 subjects in the negative cohort, and 455 randomly sampled patches extracted from locations with future lesions from 15 subjects in the positive cohort. The randomly extracted patches of the validation and test set were used to evaluate our anomaly detection approach locally. Two subjects were not part of the local evaluation on patch level because patches extracted from the location of the future lesion also contained more than 50 pixels of background and were therefore excluded.

To extract patches from locations with future lesions, the position was annotated by an expert radiologist with 25 years of experience in breast MRI but without specific training for this study, based on the first contrast-enhanced subtracted image of the visit for which the presence of the lesion was confirmed for the first time. ITK-Snap 3.8.0 was used for annotation (www.itksnap.org [34]). After intra-subject registration of visits, this manual annotation was also available for earlier examinations. See also Fig. 2 for an overview of the dataset creation.

In addition to local evaluation on the patch level, we also performed an examination-level evaluation, corresponding to a check of the entire MRI scan of a subject for a specific screening visit. For this, we applied our model to the subjects of validation and test set in a sliding window approach over the entire breast tissue. The sliding window approach was applied to all inter-visit difference images of consecutive BI-RADS 1-graded [35] visits available for a subject. As some subjects of the negative cohort provided more than one examination for evaluation, this results in 21 examinations from 11 subjects. In the positive cohort, 20 examinations from 20 subjects were used.

### Preprocessing

Preprocessing included automated registration of follow-ups to reach spatial correspondence over time, automated breast segmentation to localize breast tissue in the scans, and extraction of patches as input for anomaly detection by our DL model. 64 × 64 pixel-sized patches were extracted from voxel value difference images *I*_*i,j*_^*diff*^ = *I*_*i*_^*sub*^*—I*_*j*_^*sub*^ between screening visits *i* and *j*. *I*_*i*_^*sub*^ is the first post-contrast subtracted volume of visit *i* and *i* refers to the *i*^th^ available visit of a subject ordered according to the acquisition date. The lesion annotations were used additionally to extract patches from locations of future lesions. The number of patches extracted from a subject depended on breast size, ranging from around 200 to over 70,000 for subjects of the negative cohort and 20–40 patches of future lesion locations per subject of the positive cohort. More details on registration, segmentation, and patch extraction are provided in the Supplementary Material.

### Training a fast anomaly detection generative adversarial network (f-AnoGAN)

**Fig. 3:**
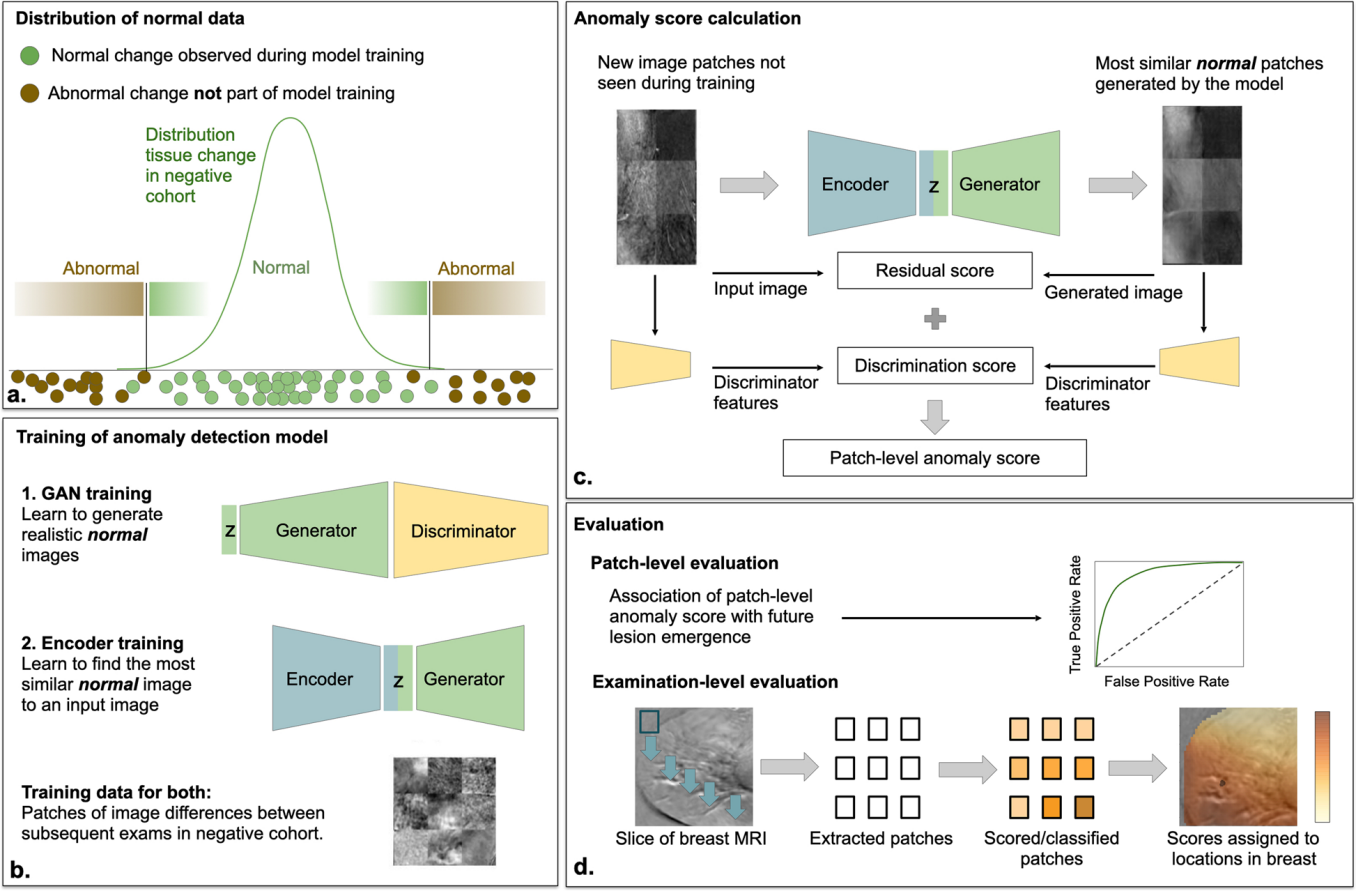
Overview of GAN-based anomaly detection and future lesion emergence prediction. **a** Tissue appearance change in negative follow-up CE-MRI scans of the breast that do not develop into breast cancer (normal data) forms a distribution in the space of different images that can be learned by a GAN model from normal cases. Anomalies are observations deviating from this distribution. **b** During training, a Generator *G*, a discriminator *D*, and an encoder *E* are trained on normal data. **c** During anomaly scoring a new image patch is processed by *E*, *G*, and *D*, resulting in an anomaly score. An anomaly score map for an entire CE-MRI volume is composed of anomaly scores evaluated in a sliding window across the entire volume. *CE-MRI* Contrast-enhanced magnetic resonance imaging, *GAN* Generative adversarial network

The extracted patches from *I*_*i,j*_^*diff*^ represent differences in voxel values of subsequent visits and therefore the change of breast tissue appearance across time. We used anomaly detection to identify abnormal changes as possible precursors of future lesions. An overview of the anomaly detection framework is presented in Fig. 3. The idea of anomaly detection is to create a baseline model that captures normal variability. Then, deviations from this model are detected as anomalies (Fig. 3a). To model the variability of normal tissue appearance change over time, we used an f-AnoGAN [36] trained on the training set consisting only of patches extracted from the negative cohort. The proposed approach builds upon preliminary work presented by Burger et al. [37], significantly extending it with respect to anomaly detection, examination-level anomaly scoring, and more patients used for training and evaluation.

The f-AnoGAN consists of a generator *G,* discriminator *D*, and encoder *E* and is trained in two steps (Fig. 3b). First, *G* is trained to generate samples following the distribution of the training set using a random noise vector **z** as input, whereas *D* is trained to assess how well the generator mimics the training data. Thereby the discriminator helps the generator to learn the training data distribution [38]. After training, G can synthesize examples that follow the distribution of normal tissue appearance change during follow-up exams. Second, the encoder *E* is trained to estimate the noise space embedding **z** of an input image **x**. Finally, the trained models can be used to process a new input image **x** as follows: the encoder E maps x to the noise space representation **z**, which is then used by G to generate a reconstructed image **y** = *G*(*E*(**x**)) which should be as similar as possible to **x**. As f-AnoGAN is only trained on examples of normal variability, the generated image **y** is a normal version of the original image **x**. If **x** shows normal variability itself, **x** and **y** will be very similar. If **x** shows an anomaly, **y** will be different from **x**. This circumstance forms the basis of our anomaly detection strategy.

### Calculating a local image anomaly score

The anomaly score measures the deviation from normal appearance change by comparing **x** and **y** and is based on two components: a residual score *L*_*R*_(**x**) and a discriminator score *L*_*D*_(**x**). The residual score measures the similarity of the original patch **x** and generated patch **y**, whereas the discriminator score measures how normal the original patch **x** is. The anomaly score is calculated as a weighted sum *L(x)* = *L*_*R*_(**x**) + *kL*_*D*_(**x**), where *k* = 0.1 is a weighting term (Fig. 3c). Details on the calculation of *L*_*R*_(**x**) and *L*_*D*_(**x**) can be found in the Supplementary Material.

### Evaluation of the local association between anomalies and future lesion emergence

For evaluating the association of the local anomaly score and the emergence of lesions in the future (Fig. 3d), we conducted bootstrapping [39] to obtain a distribution of receiver operating characteristic (ROC) curves on the test set. A single ROC curve was computed using 128 randomly selected patches from both the positive and negative cohort. This process was then repeated 10 times. ROC curves were chosen over precision-recall curves because we considered the correct classification of normal and abnormal changes equally important. Based on each single ROC curve, an optimal classification threshold was calculated using Youden’s index [40]. Then, sensitivity, specificity, and false positive rate were calculated for this threshold. Finally, we computed 95% confidence intervals for these metrics. More details on performance evaluation are provided in the Supplementary Material.

### Evaluation of an examination-level anomaly score

To get an examination-level score, we used a sliding window over the whole breast indicated by the segmentation mask (Fig. 3d) and aggregated over all voxels in both breasts. More details on how all local anomaly scores are aggregated into a single examination-level score can be found in the Supplementary Material. We evaluated the association between this examination-level score and the future occurrence of a lesion at any location by logistic regression, with the examination-level score as an independent and future lesion presence as the dependent variable. Additionally, a variable indicating the scanner type was included as an independent variable to control for possible correlations between scanner types and the anomaly score. To test for the statistical significance of the coefficient of the score, which corresponds to the significance of the association between examination-level score and future lesion, we used a Wald test with a significance level of *α* = 0.05. We also provide performance measures and bootstrapping confidence intervals for various classification thresholds of the examination-level score *T*_exam_. To additionally take the malignancy of future lesions into account, we also evaluated the model on BI-RADS 1, 2 *versus* BI-RADS 3, 4, and 5 classifications. The number of corresponding examinations are then 33 and 8, respectively. We decided to summarize BI-RADS 3 together with BI-RADS 4 and 5 lesions into one group to not further decrease the smaller class to 6.

## Results

### Anomalies predict future local lesion emergence

**Fig. 4:**
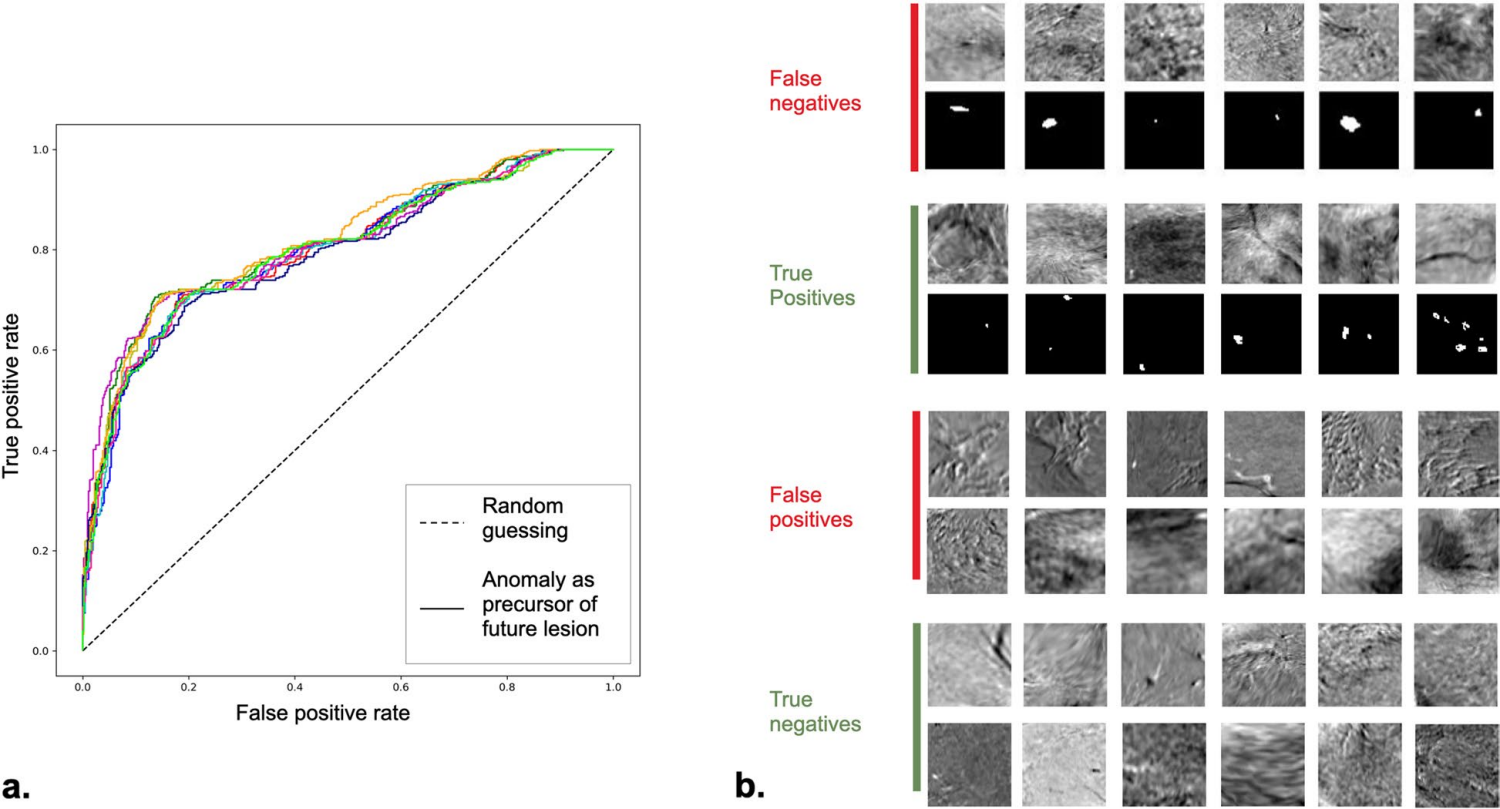
The association of anomaly scores in negative scans (BI-RADS 1) and the emergence of lesions (BI-RADS 2 and above) in future scans. **a** ROC curves for 10 randomly drawn subsamples of the test set with equal numbers of subjects from the positive and negative cohorts are shown in different colors. Each color corresponds to one of the subsamples. **b** Illustrative examples of false negatives (low anomaly, future lesion), true positives (high anomaly, future lesion), false positives (high anomaly, no future lesion), and true negatives (low anomaly, no future lesion) in the test set. *BI-RADS* Breast Imaging and Reporting Data System

We evaluated the association of an elevated anomaly score with future lesion emergence at known future lesion locations using a distribution of ROC curves and obtained a confidence interval for the area under the ROC curve of (0.80, 0.82). The ROC curves are illustrated in Fig. 4a. The calculated optimal classification threshold for the anomaly score based on each ROC curve ranged from 552.43 to 603.88; 95% confidence intervals for true positive rate (sensitivity) and false positive rate at the determined cutoffs were 69 to 71% and 14 to 18%, respectively, and 82 to 86% for mean specificity. Table 1 shows the classification threshold, sensitivity, false positive rate, specificity, and area under the curve for each ROC curve. Figure 4b shows examples of false positives and false negatives from the test set.

**Table 1:** Evaluation measures for a local association of anomaly score and future lesion occurrence

ROC curve number	AUC	Classification threshold based on ROC curve	Sensitivity	Specificity	False positive rate
1	0.81	585.14	70%	85%	0.15
2	0.80	552.43	71%	83%	0.17
3	0.82	585.14	70%	84%	0.16
4	0.81	572.91	70%	86%	0.14
5	0.81	559.86	71%	84%	0.16
6	0.82	595.71	69%	86%	0.14
7	0.81	585.14	70%	84%	0.16
8	0.80	595.71	69%	85%	0.15
9	0.80	559.86	71%	82%	0.18
10	0.82	552.43	71%	84%	0.16

*ROC* Receiver operating characteristic, *AUC* Area under the curve

At the lesion level, for 5 future lesions out of 18, less than 50% of the patches covering the future lesion location were detected as anomalous. All these five lesions were benign, and four of the five had been classified as BI-RADS 2 when discovered by a radiologist. The fifth lesion had been classified as BI-RADS 4 when discovered by a radiologist but was later identified as mastitis.

### An elevated examination-level score is associated with future lesion emergence at any breast location

**Fig. 5:**
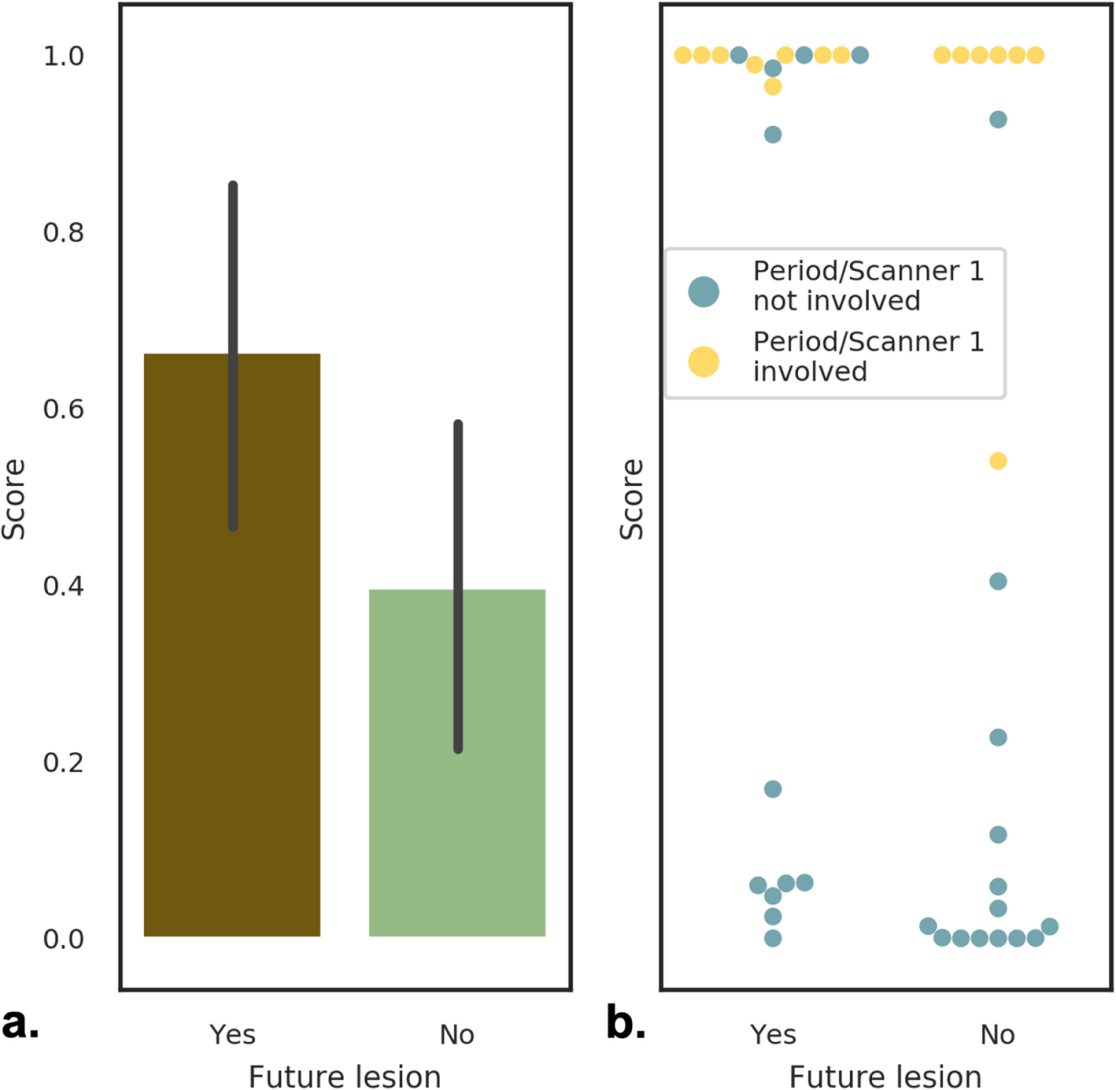
Examination-level scores for the negative and positive cohorts. **a** Mean anomaly score and standard deviation for positive examinations (future lesion: Yes) and negative examinations (future lesion: No). **b** Swarm plot displaying individual subject scores. The color indicates if one of the studies used to calculate the inter-visit difference image was acquired in Period 1 in which a different protocol and scanner were in use than in Periods 2 and 3, showing that a substantial portion of false positives falls into this category

To evaluate the association of the examination-level anomaly score with the emergence of future lesions through logistic regression we used the subjects from the validation and test set. Figure 5a shows the mean and standard deviation of examination-level scores corresponding to the mean over classified breast tissue voxels for examinations of subjects from the positive and negative cohort. The score lies by definition between 0 and 1. When predicting the emergence of a lesion from the examination-level score via logistic regression, the score’s coefficient was found to be significantly different from 0 (coefficient = 2.006, 95% confidence interval 0.057 to 4.913; *p* = 0.045).

When investigating the association of the score with future lesion emergence, we also found hints for an association between score and acquisition period. More precisely, there was an association between images acquired in Period 1 and a high examination-level score as seen in Fig. 5b. At an examination-level classification threshold *T*_*exam*_ ranging from 0.1 to 0.9 most false positives corresponded to inter-subject difference images calculated based on at least one study acquired in Period 1 (yellow dots in Fig. 5b). Since we included a scanner variable in the logistic regression model, results, nevertheless, showed that the score is a significant independent predictor of future lesion emergence. Supplementary Table 1 summarizes several performance measures for various values of *T*_*exam*_, yielding a good compromise at *T*_*exam*_ = 0.7 and *T*_*exam*_ = 0.9. When classifying BI-RADS 1, 2 *versus* BI-RADS 3, 4, and 5 with *T*_*exam*_ = 0.7, sensitivity and negative predictive value increase from 65 and 67% to 88% and 95%; however, specificity and positive predictive value drop from 67 and 65% to 61% and 38%.

## Discussion

Our study shows that anomalous change of CE-MRI between negative screens is associated with future lesion emergence in high-risk women. Anomalies can be detected by a generative adversarial network (GAN) model trained on CE-MRI screens of women who remain BI-RADS 1 throughout the observation period. We evaluated the method on a large high-risk screening study cohort, achieving a mean area under the curve (AUC) of 0.804 in predicting future lesion emergence based on the current local anomaly score in negative CE-MRI screens. The score predicts a transition from BI-RADS 1 to BI-RADS 2, 3, 4, or 5 at a later time point. Additionally, we evaluated if the summary of anomaly scores in the entire breast is associated with future lesion emergence at any location and observed a significant association (*p* = 0.045).

This suggests that anomaly detection approaches may serve as a basis for dynamic risk scoring. In the context of high-risk screening, anomalies with an elevated risk of future lesion emergence could be used to adjust screening intervals, or as a support for radiologists assessing a screening scan. The paper forms a basis for future research to develop corresponding adaptive individualized screening strategies.

Usually, a prophylactic bilateral mastectomy is offered to high-risk women as soon as their risk status has been determined but many women decline surgery at that point [41]. Through dynamic risk profiling the surgery could be offered to women exactly when the risk of cancer development in the following year is even more elevated due to already present anomalies. This would allow a high-risk woman to avoid surgery as long as possible and at the same time prevent her from becoming a cancer patient. A prerequisite for risk adjustment is the identification of precursor appearance signatures at the location of future lesions. A prerequisite for risk adjustment is the identification of precursor appearance signatures at the location of future lesions. Additionally, successful automated detection of anomalies in breast tissue change between subsequent screening visits would save resources for radiologists, who currently have to spend their efforts on visually comparing MRI scans. Despite being time-consuming, a visual comparison is also exhausting and leads to fatigue potentially accompanied with increased susceptibility to reading errors. Reliable automated detection of anomalies could therefore reduce the workload of radiologists and consequently acquire more of their resources for assessing suspicious cases.

Deep learning models have an increasingly important role in the detection and quantification of findings in medical imaging data [18]. The majority of approaches demonstrates that models can be successfully trained by supervision based on large numbers of pairs of available images and labels, such as lesion segmentation [42]. This is feasible for the detection of well-described lesions that can be annotated by experts. However, it does not allow for the discovery of yet unknown predictive signatures in imaging data, since annotation is not possible, or the number of positive subjects is comparably low as is the case in screening scenarios. Anomaly detection approaches such as f-AnoGAN [36] offer to discover anomalies in the change of breast tissue during follow-up, that is linked to future lesion emergence, without the need for extensive annotation for training.

Models based on f-AnoGAN capture complex appearance variability in the negative cohort well and can detect precursor signatures of future lesions without the need for annotation. Previously, GANs have been used for anomaly detection in medical images [36, 43, 44]. In contrast to these results for detecting present lesions, here we use GANs to detect precursor signatures of lesions that have not yet emerged. To our knowledge, this study is the first to propose a GAN-based anomaly detection strategy to predict future lesions in a cohort of high-risk women using CE-MRI scans acquired during screening. We demonstrated that precursor signatures of lesions that emerge at a later time point can be identified by unsupervised machine learning, performing training only on the negative cohort.

Our model, trained on follow-up CE-MRI exams of women at high BC risk who do not develop BC, captures the normal variability of longitudinal change in the appearance of CE-MRI of the breast. Applying this model to new imaging data derives an anomaly score that quantifies the deviation of the observed longitudinal appearance change from normal variability represented in the model. By looking at tissue changes over time, our approach therefore mimics the approach of radiologists when screening images.

In our analysis of cases where the presence of an anomaly misclassified the future emergence of lesions, we identified two primary factors as possible causes. First, the longitudinal dataset collected over a period of 17 years was acquired by a consecutive series of three different scanner parameters. A substantial number of misclassifications were linked to a scanner switch occurring between compared examinations, for various values of the classification threshold *T*_*exam*_ for the examination-level score. This suggests unsurprisingly that scanner type and protocol are relevant for an appearance change. In our experiments, the anomaly score was nevertheless an independent predictor for future lesion emergence, with a compromise of sensitivity, specificity, and negative and positive predictive value at *T*_*exam*_ = 0.7 and *T*_*exam*_ = 0.9. The second factor that mainly produced false negatives (low anomaly score, despite a lesion emerging at a later time point) was exclusively benign lesions. Classifying BI-RADS 1 or 2 *versus* BI-RADS 3, 4, or 5 therefore showed a raise in sensitivity and negative predictive value. This was, however, also accompanied with a drop of the positive predictive value, mainly caused by false positives that were either related to the scanning period or BI-RADS 2 lesions. The purpose of this study was, however, to predict lesions of any type.

Although the focus of our study is on future lesion prediction, for clinical applicability the preprocessing is also relevant. Twenty-two subjects were removed from the dataset due to unsatisfactory preprocessing results. Registration of follow-up data was to be the least problematic and only one subject was lost due to misregistration of a screening visit acquired during the oldest acquisition period, period 1. Too small breast size for patch extraction affected eight subjects, but was mainly a limitation, for screening visits acquired during Period 2. For scans with higher resolution, such as those in Period 3, only one subject was excluded. A patch size of 64 × 64 might therefore be feasible for most subjects at the resolution of current MRI protocols. Furthermore, the patch size used in the model could be changed, if a smaller patch size should be more feasible. The preprocessing step leading to most exclusions (*n* = 13 subjects) was segmentation, due to unsatisfactory segmentation results. U-nets [42], which are the current state-of-the-art for segmentation, could improve the segmentation of breast tissue. However, they first would need to be trained on already manually annotated MRI scans.

One main limitation of this study is the small sample size with only 64 subjects on the patient level. Apart from that, we evaluated our approach on the patch level, yielding a sample size of 104,531 patches of normal tissue change and 455 patches of future lesion locations. Furthermore, although our method mimics the approach of radiologists when screening imaging data by searching for differences between subsequent screening visits, our model requires at least two consecutive studies reflecting the high-risk screening population, where regular yearly CE-MRI scans of the breast are performed. This makes it inapplicable to women on their first visit. Another limitation of the study is the scanner bias when evaluating our examination-level score. Heterogeneity in medical data, such as the use of different scanners and acquisition protocols, is a common problem for computational image analysis. Nevertheless, our anomaly score was associated with future lesions regardless of this factor. We aggregated BI-RADS 2, 3, 4, or 5 scores to a single class (> BI-RADS 1), without differentiating malignancy. However, we performed a post hoc analysis of the results and investigated the possible malignancy of lesions that were not successfully predicted and found that they were all benign. Even though the study was conducted on 1.5-T CE-MRI data, we expect the results to be transferable directly also to higher field strength scanners such as 3 T. Increased image quality might further facilitate the identification of precursor image signatures.

In summary, anomaly detection is a feasible approach to identify precursor signatures of future lesion emergence in CE-MRI of high-risk women, which might form the basis for adjusting individual screening intervals. Training a representative model of normal variability can detect signatures that are not yet well defined, without the need for annotated examples with lesions. Therefore, our approach is in principle also applicable to CE-MRI of women at normal risk. However, further validation is required to assess the feasibility of implementing corresponding adaptive screening strategies on normal-risk populations. In the context of high-risk screening, the next steps will be the investigation of prediction accuracy for benign and malignant lesions, as well as the influence of background enhancement and breast density on our anomaly score.

## Supplementary Information

**Additional file 1.**

## Data Availability

The datasets generated and/or analyzed during the current study are not publicly available due to the need for a legal contract regulating data protection but are available from the corresponding author on reasonable request.

## Abbreviations

AUC: Area under the curve
BC: Breast cancer
CE-MRI: Contrast-enhanced magnetic resonance imaging
DL: Deep learning
f-AnoGAN: Fast anomaly detection generative adversarial network
GAN: Generative adversarial network
*I*_*i*__*,j*_^*diff*^: Voxel value difference image of *I*_*i*_^*sub*^ and *I*_*j*_^*sub*^
*I*_*i*_^*sub*^: First post-contrast subtraction image of the *i*th available screening visit of a subject
ROC: Receiver operating characteristic
