# Supplementary material for: Deep learning for predicting future lesion emergence in high-risk breast MRI screening: a feasibility study
Source: Eur Radiol Exp. 2023 Jun 7;7:32. doi: 10.1186/s41747-023-00343-y (PMC10244308; doi:10.1186/s41747-023-00343-y)
Supplement: Supplementary file 1 — Additional file 1. [file 41747_2023_343_MOESM1_ESM.pdf]

Deep learning for predicting future lesion emergence in high-risk breast MRI screening: a feasibility study

Supplementary Material – Table of contents:

- Methods Details
- Supplementary Results
- CLAIM checklist
- Supplementary References

Methods Details

Preprocessing

Registration of follow-up volumes for spatial correspondence over time. First, we performed automated registration of MRI sequences acquired during follow-up visits to establish correspondence and enable the tracking of appearance changes over time. While volumes of the same visit were already co-registered to each other by the device, follow-up volumes across visits of the same patient were lacking registration. Automatic registration was conducted using bias field correction, followed by an affine and a non-rigid diffeomorphic transformation, based on the pre-contrast images of each visit. The last volume in the respective follow-up series thereby served as fixed image in the registration process. Affine transformations are calculated using ANTS registration software [1] and mutual information as similarity criterion after initial alignment of the centres of mass of moving and fixed image. The non-rigid diffeomorphic transformations are calculated using Ezys [2] and normalized mutual information. Both registration algorithms use a coarse-to-fine-scheme in 3 steps. Non-rigid diffeomorphic transformations with Ezys are calculated using the symmetry option, which

averages direct and indirect registration transformation. The quality of registration results was verified by visual inspection. Unsatisfactory registration results due to spatial mismatch of breast tissue were either excluded or recalculated using a different fixed image, i.e., either the volume for which lesion occurrence should be predicted or, for control subjects, the last consecutive volume with BIRADS 1.

Segmentation of breast tissue. After registration, the breast was segmented automatically using an atlas-based segmentation technique. Here we used an atlas-based segmentation approach as proposed by Vogl et al [3]. Nine breast templates, covering a range of breast sizes and shapes and consisting of an image and a corresponding breast annotation mask each, were registered to the last volume in the respective follow-up series of each subject. An affine transformation was followed by a non-rigid diffeomorphic transformation using the same software and settings as for the registration pipeline [4]. The best breast mask was chosen after registration based on the dice coefficient calculated between the thresholded pre-contrast image and the breast masks. The threshold was 0.1 and taken over from Vogl et al [3]. The corresponding breast mask was then used as segmentation and verified by visual inspection.

Patch extraction. After registration and segmentation, the post-contrast subtracted volume I_i^{sub} of each visit was calculated as the difference between the pre-contrast volume and the first post-contrast volume, where i refers to the ith available visit of a subject ordered according to acquisition date. The voxel value difference of registered follow-up images formed images $I_{i,j}^{diff} = I_i^{sub} - I_j^{sub}$. We then extracted 64 x 64 pixel-sized patches from the difference images $I_{i,j}^{diff}$. Within each slice, we extracted patches at random positions within the breast mask. To ensure that they contained a sufficient amount of breast tissue, we only allowed 50 background-pixels within a patch. For that, we first created a pool of patch centres so that the full patch would still be completely inside the MRI image. Then, we chose patches based on the theoretically possible patch centres in a slice maximally 5000 times and accepted the patch when the number of background voxels did not exceed 50. If 1/500 of all theoretically possible patches had been accepted, we stopped the extraction from the current slice before 5000.

The lesion annotations were used additionally to extract patches from locations of future lesions. After completed extraction, the pixel values of patches were normalized patch-wisely by shifting their range to [-1,1].

Anomaly detection model

Details on residual and discriminator score calculation. Residual score $L_R(\mathbf{x})$ is computed by the difference between observed and generated images. To also take differences in the gray value ranges between different MR images of subjects into account, we scaled the gray values of the input and generated image to match again the original gray value distribution after MRI acquisition. This is performed by the scaling function $s(\mathbf{x})=(\mathbf{x}/2+0.5)(x_{max}-x_{min})+x_{min}$, where x_{max} and x_{min} are the highest and lowest gray value of $\mathbf{x}$. The residual score is then $L_R(\mathbf{x})1/n\ ||s(\mathbf{x})-G(E(s(\mathbf{x})))||^2$, where $n=64^2$ is the number of pixels of $\mathbf{x}$. For discriminator score $L_D(\mathbf{x})$, the discriminator D is used to obtain a feature representation $f(\mathbf{x})$ and $f(G(E(\mathbf{x})))$ of both the observed and generated image, whereby the features $f(.)$ correspond to the output of the 4th layer of D. The difference between these feature representations yields the discriminator score $L_D(\mathbf{x})=1/n_d\ ||f(\mathbf{x})-f(G(E(\mathbf{x})))||^2$, where n_d is the number of features calculated by $f(.)$.

Evaluation

Role of validation and test set for evaluating local associations between anomalies and future lesions. For evaluating the association of anomalies and future lesions, we used the validation set to compare different training settings of the model and the test set for final evaluation of the best training setting. Both sets contained 100 times more normal patches than patches extracted from locations of future lesions. Therefore, positive and negative cohorts for each set were randomly subsampled to match the number of normal patches and patches extracted from locations of future lesions for calculating ROC curves.

On the validation set, we compared the effect of 2 different losses for training encoder E on the prediction capacity. The first one is the loss formulation as given in Schlegel et al [5], which also corresponds to the definition of their anomaly score. The second loss function used for training corresponds to our definition of the anomaly score and includes the rescaling of the gray values of a patch. As there was, however, no clear difference in performance, we decided to stick to the original formulation and evaluate it on the test set in terms of sensitivity and precision to predict future lesions.

Examination-level anomaly score calculation. Obtaining a risk score for an entire examination is important to decide on the optimal interval between current and next screening visit. It should help to reduce the scanning of negative subjects, while reducing the risk of cancer growth between screening visits at the same time. To get an examination-level score, we first used a sliding window of size 64 x 64 over the whole breast indicated by the segmentation mask. The sliding window thereby moved over each transverse slice with a step size of (3, 3). Next, an anomaly score was assigned to each patch. Based on the score, patches were classified as showing normal tissue if the score was below a threshold T and as anomalous above T. T was thereby determined based on the evaluation of local associations between anomalies and future lesion emergence, as the smallest ROC-based classification threshold. Based on the 10 ROC curves calculated during local evaluation, the lowest threshold determined was T = 552.43. The assigned classification labels were coded numerically as 0 and 1, respectively, and the value was assigned to each pixel of the patch. The patch-wise results were then assigned to the corresponding positions in the three-dimensional breast. At pixel positions where patches overlapped, patch values were averaged. A score on examination-level was then obtained by averaging over all voxels in both breasts.

Supplementary Results

Supplementary Table 1. Evaluation measures with confidence intervals for association of examination-level anomaly score and future lesion occurrence. Confidence intervals calculated via bootstrapping are stable, since examination-level scores of subjects are almost binary (compare Figure 5 in the main document).

Classification threshold T_{exam}	Sensitivity	Specificity	Positive predictive value	Negative predictive value
0.1	70% (70%, 70%)	48% (48%, 48%)	56% (56%, 56%)	63% (63%, 63%)
0.3	65% (65%, 65%)	57% (57%, 57%)	59% (59%, 59%)	63% (63%, 63%)
0.5	65% (65%, 65%)	62% (62%, 62%)	62% (62%, 62%)	65% (65%, 65%)
0.7	65% (65%, 65%)	67% (67%,67%)	65% (65%, 65%)	67% (67%, 67%)
0.9	65% (65%, 65%)	67% (67%, 67%)	65% (65%, 65%)	67% (67%, 67%)

CLAIM: Checklist for Artificial Intelligence in Medical Imaging [6]

Section / Topic	No.	Item	Comment
**TITLE / ABSTRACT**			
	**1**	Identification as a study of AI methodology, specifying the category of technology used (e.g., deep learning)	"Deep Learning" is in the title"
	**2**	Structured summary of study design, methods, results, and conclusions	abstract follows the structure fixed by the journal, number of patches / examinations / subjects are given, summary of statistical analysis is provided
INTRODUCTION			
	**3**	Scientific and clinical background, including the intended use and clinical role of the AI approach	clinical background: paragraph 1-2,<br>clinical role and intended use: paragraph 3-4
	**4**	Study objectives and hypotheses	paragraph 5
METHODS			
Study Design	**5**	Prospective or retrospective study	abstract: 1st sentence in Methods,<br>main text: 1st sentence in paragraph "Data Collection"
	**6**	Study goal, such as model creation, exploratory study, feasibility study, non-inferiority trial	included in title and paragraph "Study overview"
Data	**7**	Data sources	paragraph "Data Collection"
	**8**	Eligibility criteria: how, where, and when potentially eligible participants or studies were identified (e.g., symptoms, results from previous tests, inclusion in registry, patient-care setting, location, dates)	paragraph "Data Collection"
	**9**	Data pre-processing steps	main text: paragraph "Preprocessing"

			Supplementary Material: “Preprocessing”
	**10**	Selection of data subsets, if applicable	Supplementary Material: paragraph “Patch extraction”
	**11**	Definitions of data elements, with references to Common Data Elements	main text: paragraphs “Evaluation of the local association between anomaly and future lesion emergence” and “Evaluation of an examination-level anomaly score”
	**12**	De-identification methods	anonymized data: 2nd sentence in “Data Collection”
	**13**	How missing data were handled	not applicable
Ground Truth	**14**	Definition of ground truth reference standard, in sufficient detail to allow replication	BI-RADS and lesions annotations: paragraphs “Data Collection” and 2nd subparagraph of “Data preparation”
	**15**	Rationale for choosing the reference standard (if alternatives exist)	not applicable
	**16**	Source of ground-truth annotations; qualifications and preparation of annotators	an expert radiologist: 2nd subparagraph of “Data preparation”
	**17**	Annotation tools	2nd subparagraph of “Data preparation”
	**18**	Measurement of inter- and intrarater variability; methods to mitigate variability and/or resolve discrepancies	not provided
Data Partitions	**19**	Intended sample size and how it was determined	we worked with the data that survived our inclusion criteria
	**20**	How data were assigned to partitions; specify proportions	paragraph “Data Preparation”
	**21**	Level at which partitions are disjoint (e.g., image, study, patient, institution)	paragraph “Data Preparation”
Model	**22**	Detailed description of model, including inputs, outputs, all intermediate layers and connections	main text: paragraphs “Training f-AnoGAN for

			anomaly detection" and "Calculating a local image anomaly score"<br>Supplementary Material: "Anomaly detection model"<br>details on model architecture in f-AnoGAN-paper [5]
	**23**	Software libraries, frameworks, and packages	f-AnoGAN-paper [5]
	**24**	Initialization of model parameters (e.g., randomization, transfer learning)	f-AnoGAN-paper [5]
Training	**25**	Details of training approach, including data augmentation, hyperparameters, number of models trained	f-AnoGAN-paper [5]
	**26**	Method of selecting the final model	Supplementary Material: paragraph "Role of validation and test set for evaluating local associations between anomalies and future lesions"
	**27**	Ensembling techniques, if applicable	not applicable
Evaluation	**28**	Metrics of model performance	paragraphs "Evaluation of the local association between anomaly and future lesion emergence" and "Evaluation of an examination-level anomaly score"
	**29**	Statistical measures of significance and uncertainty (e.g., confidence intervals)	paragraphs "Evaluation of the local association between anomaly and future lesion emergence" and "Evaluation of an examination-level anomaly score"
	**30**	Robustness or sensitivity analysis	partly provided - Supplementary Table 1
	**31**	Methods for explainability or interpretability (e.g., saliency maps), and how they were validated	not provided

	**32**	Validation or testing on external data	not provided
RESULTS			
Data	**33**	Flow of participants or cases, using a diagram to indicate inclusion and exclusion	Figure 2
	**34**	Demographic and clinical characteristics of cases in each partition	not provided
Model performance	**35**	Performance metrics for optimal model(s) on all data partitions	Table 1
	**36**	Estimates of diagnostic accuracy and their precision (such as 95% confidence intervals)	main text: paragraph “Anomalies predict future local lesion emergence”<br>Supplementary Table 1
	**37**	Failure analysis of incorrectly classified cases	2nd subparagraph of “Anomalies predict future local lesion emergence”
DISCUSSION			
	**38**	Study limitations, including potential bias, statistical uncertainty, and generalizability	summary and context: paragraph 1: 1st subparagraph, paragraphs 2, 3<br>limitations: paragraph 4, 6
	**39**	Implications for practice, including the intended use and/or clinical role	paragraph 1: 2nd subparagraph, paragraphs 5, 7
OTHER INFORMATION			
	**40**	Registration number and name of registry	not applicable
	**41**	Where the full study protocol can be accessed	all information provided in paper and Supplementary Material
	**42**	Sources of funding and other support; role of funders	Declarations Section

Supplementary References

1. Avants BB, Tustison NJ, Song G, et al (2011) A reproducible evaluation of ANTs similarity metric performance in brain image registration. Neuroimage 54:2033–2044
2. Gruslys A, Acosta-Cabronero J, Nestor PJ, et al (2014) A new fast accurate nonlinear medical image registration program including surface preserving regularization. IEEE Trans Med Imaging 33:2118–2127
3. Vogl W-D, Helbich KPTH, Bickel H, Grabner G, Bogner W, Gruber S, Bago-Horvath Z, Dubsky P, Langs G (2019) Automatic segmentation and classification of breast lesions through identification of informative multiparametric pet/mri features. Eur Radiol Exp 3. https://doi.org/10.1186/s41747-019-0096-3
4. Burger B, Bernathova M, Helbich T, et al (2020) AI-based prediction of lesion occurrence in high-risk women based on anomalies detected in follow-up examinations. In: Bosmans H, Marshall N, Van Ongeval C (eds) 5th International Workshop on Breast Imaging (IWBI2020). Proceedings of SPIE, vol 11513. International Society for Optics and Photonics
5. Schlegl T, Seeböck P, Waldstein SM, et al (2019) f-AnoGAN: Fast unsupervised anomaly detection with generative adversarial networks. Med Image Anal 54:30–44
6. Mongan J, Moy L, Kahn CE Jr (2020) Checklist for Artificial Intelligence in Medical Imaging (CLAIM): A Guide for Authors and Reviewers. Radiol Artif Intell 2:e200029. https://doi.org/10.1148/ryai.2020200029
